# The Association of Common Functional Polymorphisms in Mir-146a and Mir-196a2 and Hepatocellular Carcinoma Risk: Evidence from a Meta-Analysis

**DOI:** 10.1097/MD.0000000000000252

**Published:** 2014-12-02

**Authors:** Qiliu Peng, Shan Li, Xianjun Lao, Zhiping Chen, Ruolin Li, Yan Deng, Xue Qin

**Affiliations:** From the Department of Clinical Laboratory, First Affiliated Hospital of Guangxi Medical University, Nanning, Guangxi, China (QP, SL, XL, YD, XQ); Department of Occupational Health and Environmental Health, School of Public Health at Guangxi Medical University, Nanning, Guangxi, China (ZC); and Department of Medicine Research, First Affiliated Hospital of Guangxi Medical University, Nanning, Guangxi, China (RL).

## Abstract

MicroRNAs (miRNAs) are small non-coding RNA molecules that function as tumor suppressors or oncogenes. Single nucleotide polymorphisms (SNPs) located in the miRNAs influence the function of mature miRNAs and may contribute to cancer development. Studies investigating the association between miR-146a rs2910164 and miR-196a2 rs11614913 polymorphisms and hepatocellular carcinoma (HCC) risk reported inconsistent results. We performed a meta-analysis of all available studies to summarize this situation.

Eligible studies were identified by search of electronic databases including PubMed, Embase, and Cochrane library for the period up to August 2014. The association of miR-146a rs2910164 and miR-196a2 rs11614913 polymorphisms and HCC risk was assessed by odds ratios (ORs) together with their 95% confidence intervals (CIs).

Finally, a total of 12 studies with 4171 cases and 4901 controls were included for miR-146a rs2910164 polymorphism and 10 studies with 4687 cases and 4990 controls were available for miR-196a2 rs11614913 polymorphism. With respect to miR-146a rs2910164 polymorphism, statistical significant increased HCC risk was found when all studies were pooled into the meta-analysis (GG+CG vs CC: OR = 1.097, 95% CI 1.005–1.197, *P* = 0.037). In subgroup analyses by ethnicity, source of control, and HWE in controls, significant increase of HCC risk was found in Asians, population-based studies, and studies consistent with HWE, but not in Caucasians, hospital-based studies, and studies inconsistent with HWE. With respect to miR-196a2 rs11614913 polymorphism, no significant association with HCC risk was found in the overall and subgroup analyses.

The results suggest that the miR-146a rs2910164 polymorphism contributes to increased HCC susceptibility, especially in Asian populations. Further large and well-designed studies are required to validate this association.

## INTRODUCTION

Hepatocellular carcinoma (HCC), as the most frequent primary cancer of the liver, is the fifth most common cancer and the third leading cause of cancer-related deaths worldwide.^1^ The estimated annual number of cases exceeds 500,000, with a mean annual incidence of around 3–4%.^1^ Patients with HCC have a poor prognosis, with a 5-year survival rate of 5% in developing countries because of the lack of effective therapy in most patients.^2^ Etiologically, carcinogenesis of HCC is a complex, multistep, and multifactor process, in which many factors are implicated.^3^ It has been well established that major risk factors for the development of HCC are chronic infection with hepatitis B virus (HBV) or hepatitis C virus (HCV), liver cirrhosis, habitual alcohol abuse, and exposure to aflatoxin B1.^4^ However, most subjects with these environmental risk factors never develop HCC while many HCC cases develop among individuals without the above risk factors, suggesting that other factors such as genetic factor also play an important role in hepatocellular carcinogenesis.

MicroRNAs (miRNAs) are a group of small non-coding RNA molecules that regulate gene expression through complementary base pairing with thousands of messenger RNAs (mRNAs) and are demonstrated to play an important role in transcriptional and translational regulation.^5^ MiRNAs elicit their effects via imperfectly binding to the 3′untranslated region (3′UTR) of the target mRNAs, leading to either degradation of mRNAs or repression of protein translation.^6^ Gene regulation by miRNA has been involved in a diverse range of biological and pathologic processes, and miRNAs have been demonstrated to regulate the expression levels of major cancer-related genes.^7^ Emerging evidence has indicated the important influence of miRNAs on tumor invasion and metastasis through action as metastasis promoting genes or metastasis suppressor genes.^8^ More importantly, some recent studies have revealed that miRNAs participate in carcinogenesis as tumor suppressors or oncogenes by regulating the expression of their target genes.^7,8^

Single nucleotide polymorphisms (SNPs) or mutations in miRNA sequence may alter miRNA expression and/or maturation, and may also change the regulatory effect of miRNA to their target genes.^9,10^ MiR-146a rs2910164 and miR-196a2 rs11614913 were the two most common studied SNPs in miRNA. MiR-146a rs2910164 polymorphism is located at position +60 relative to the first nucleotide of pre-miR-146a with a C to G change in the passenger strand, which results in a change from C:U pair to G:U mismatch in the stem structure of miR-146a precursor and a reduced production of mature miR-146a.^11^ MiR-196a2 is composed of 2 different mature miRNAs (miR-196a-3P and miR-196a-5P) which were processed from the same stem-loop.^12^ The expression of 137 cancer-related transcripts could be significantly altered after the introduction of pre-miR-196a.^13^ Rs11614913 is located in the mature sequence of miR-196a-3P and could lead to less efficient processing of the miRNA precursor to its mature form, as well as reduced capacity to regulate target genes.^14^

In light of the important biological effects of the two miRNA SNPs, emerging epidemiological studies have been conducted to investigate the association of miR-146a rs2910164 and miR-196a2 rs11614913 polymorphisms with HCC risk, but the results remain inconsistent and underpowered. Some studies suggested that the two SNPs in miRNAs were significantly correlated with HCC susceptibility.^15–17^ However, other studies did not support such an association.^18–20^ A meta-analysis by Xu et al^21^ on this issue suggested that the miR-146a rs2910164 polymorphism was associated with a decreased HCC risk especially among Asians, while the miR-196a2 rs11614913 polymorphism was correlated with an increased HCC risk among Caucasians. However, the evidence was limited because only 7 studies were included for miR-146a rs2910164 polymorphism and 5 studies were for miR-146a rs2910164 polymorphism. In addition, the source of heterogeneity was not extensively explored in this study. As many new studies with relatively large sample size emerging,^20,22–28^ to provide the most comprehensive assessment of the association between the miR-146a rs2910164 and miR-196a2 rs11614913 polymorphisms and HCC risk, we performed an updated meta-analysis of all available studies.

## MATERIALS AND METHODS

### Literature Search

We conducted a comprehensive literature search in PubMed, EMBASE, and Cochrane library up to August 01, 2014 using the following search strategy: (“hepatocellular carcinoma,” “HCC,” or “liver cancer”) and (“miR-146a” or “miR-196a2”). There was no restriction on sample size, time period, language, population, or type of report. All eligible studies were retrieved and their references were checked for other relevant studies. In addition, we also used the “Related Articles” function in PubMed to search for other potential relevant articles. When several studies reported on the same or overlapping data, we chose the most recent or largest population. The meta-analysis was performed according to the proposal of Meta-analysis of Observational Studies in Epidemiology group (MOOSE).^29^

### Selection Criteria

Studies included in the meta-analysis were required to meet the following criteria: (1) Case–control studies which evaluated the association between miR-146a rs2910164 and/or miR-196a2 rs11614913 polymorphisms and HCC risk; (2) used an unrelated case–control design; (3) presented an odds ratio (OR) together with 95% confidence interval (CI) or other information for estimating OR (95% CI); and (4) the control group did not contain malignant tumor patients. Conference abstracts, meta-analyses, case reports, review articles, editorials, and letters were excluded.

### Data Extraction

Two authors (Shan Li and Qiliu Peng) independently extracted information from all eligible studies according to the inclusion criteria listed above. Data extracted from the qualified studies including the first author, publication year, country of origin, ethnicity, genotyping method, source of control, matching variables, HCC diagnosis, and total number of cases and controls and genotype frequencies of cases and controls. When a study did not state the ethnic descendent or if it was impossible to separate participants according to such phenotype, the group reported was categorized as “Mixed/other.” The two authors checked the data extraction results and reached consensus on all of the items.

### Statistical Analysis

The strength of the association between miR-146a rs2910164 and miR-196a2 rs11614913 polymorphisms and HCC risk was assessed by ORs together with 95% CIs. The significance of the pooled ORs was determined by *Z* test and the *P* values <0.05 were considered significant. We evaluated the miR-146a rs2910164 and miR-196a2 rs11614913 polymorphisms and HCC risk using codominant models, recessive model, and dominant model.

The Chi-square-based *Q* test was used to assess the statistical heterogeneity among studies. If the result of the *Q* test was *P*_*Q*_ < 0.10, suggesting the existence of heterogeneity, the pooled ORs were calculated using the random-effects model (the DerSimonian and Laird method).^30^ Otherwise, when the result of the *Q* test was *P*_*Q*_ ≥ 0.1, indicating the absence of heterogeneity, the fixed-effects model (the Mantel–Haenszel method) was applied.^31^ Logistic metaregression and subgroup analyses were used to explore the sources of heterogeneity among studies. The following parameters were included as covariates in the metaregression analysis: ethnicity (Asians vs Caucasians), genotyping methods (PCR-RFLP vs not PCR-RFLP), source of controls (HB vs PB), and Hardy–Weinberg equilibrium (HWE) status (Yes vs No). Subgroup analyses were performed according to ethnicity, source of control, and HWE status. Galbraith plots analysis was performed to further explore the source of heterogeneity.

Sensitivity analysis was performed by sequentially excluded the individual studies to assess the robustness of the results. Begg's funnel plot and Egger's regression asymmetry test were performed to evaluate the publication bias. If the publication bias presented, the Duval and Tweedie non-parametric “trim and fill” method was applied to adjust for it.^32^ The distribution of the genotypes in the control group was tested for HWE using a goodness-of-fit Chi-square test. All analyses were performed using Stata software, version 12.0 (Stata Corp., College Station, TX). All *P* values were two-sided. To ensure the reliability and the accuracy of the results, 2 authors entered the data into the statistical software programs independently with the same results.

## RESULTS

### Study Characteristics

Based on the search strategy, 43 records were found, but only 18 full-text studies were preliminarily identified for further detailed evaluation after screening the titles and abstracts. According to the exclusion criteria, 2 studies were excluded including 1 presenting insufficient data for calculating OR and 95% CI,^33^ and 1 was a meta-analysis.^21^ Manual search of references cited in the retrieval studies identified 1 additional study.^18^ As a result, a total of 17 studies met the inclusion criteria for the meta-analysis.^11,15–20,22–28,34–36^ The main parameters of the studies are presented in Table 1. Among them, a total of 12 studies with 4171 cases and 4901 controls were included for miR-146a rs2910164 polymorphism and 10 studies including 4687 cases and 4990 controls were available for miR-196a2 rs11614913 polymorphism. The sample size in these studies varied considerably, ranging from 200 to 2026 individuals. Of all the eligible studies, 11 were conducted in Asians and 1 was in Caucasians for miR-146a rs2910164 polymorphism; 9 were conducted in Asians and 1 was in Caucasians for miR-196a2 rs11614913 polymorphism. One study in the present meta-analysis did not provide definite criteria for the HCC diagnosis.^36^ The genotype distributions of the controls in 3 studies were inconsistent with HWE for miR-146a rs2910164 polymorphism^23,24,27^ and 2 were inconsistent with HWE for miR-196a2 rs11614913 polymorphism.^24,27^

**Table 1:**
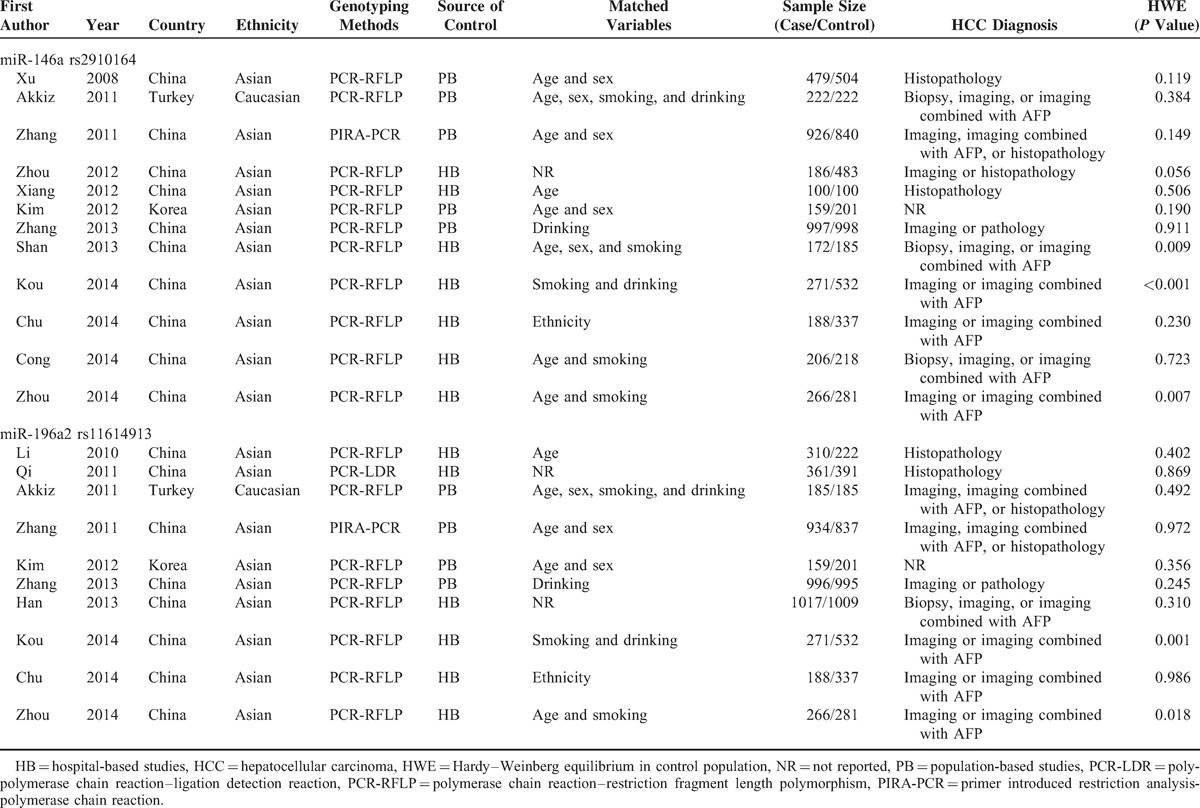
Characteristics of the Included Studies

### Meta-Analysis Results

**FIGURE 1:**
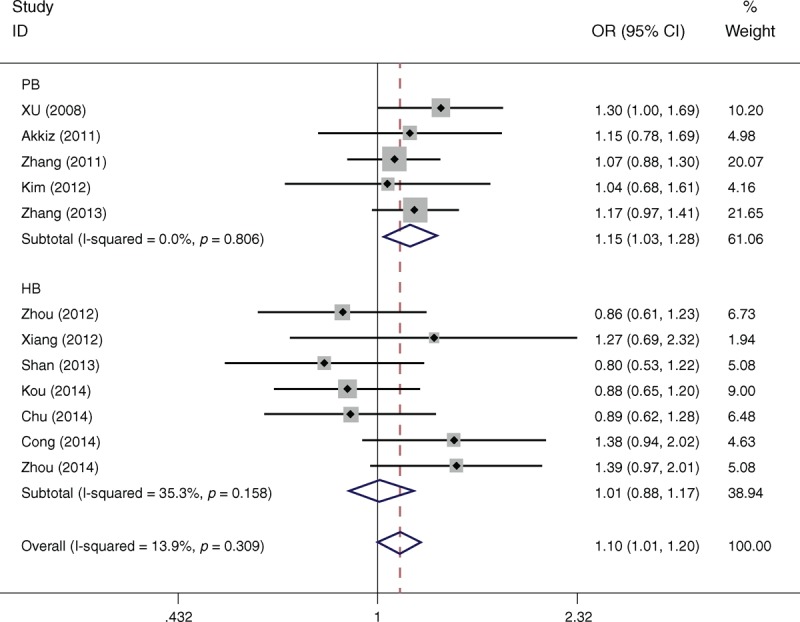
Forest plots of miR-146a rs2910164 polymorphism and HCC risk in subgroup analysis by source of controls using a fixed-effect model (dominant model GG+CG vs CC).

Table 2 lists the main results of meta-analysis of miR-146a rs2910164 polymorphism and HCC risk. Overall, significant increased HCC risk was found when all studies were pooled into the meta-analysis (GG+CG vs CC: OR = 1.097, 95% CI 1.005–1.197, *P* = 0.037). In subgroup analysis by ethnicity, significant increased HCC risk was found in Asians (GG+CG vs CC: OR = 1.194, 95% CI 1.101–1.297, *P* = 0.010), but not in Caucasians. In subgroup analysis according to source of control, significant increased HCC risk was found in population-based studies (CG vs CC: OR = 1.154, 95% CI 1.028–1.297, *P* = 0.016; GG+CG vs CC: OR = 1.150, 95% CI 1.030–1.283, *P* = 0.013; Figure 1), but not in hospital-based studies. When stratified by HWE status, significant increased HCC risk was observed in studies consistent with HWE (CG vs CC: OR = 1.114, 95% CI 1.086–1.333, *P* = 0.008; GG+CG vs CC: OR = 1.121, 95% CI 1.018–1.234, *P* = 0.012), but not in studies inconsistent with HWE.

**Table 2:**
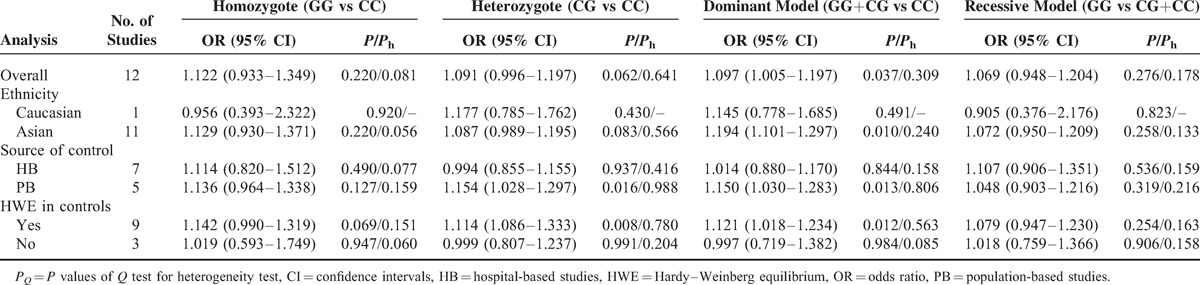
Meta-Analysis of miR-146a rs2910164 Polymorphism and HCC Risk

**FIGURE 2:**
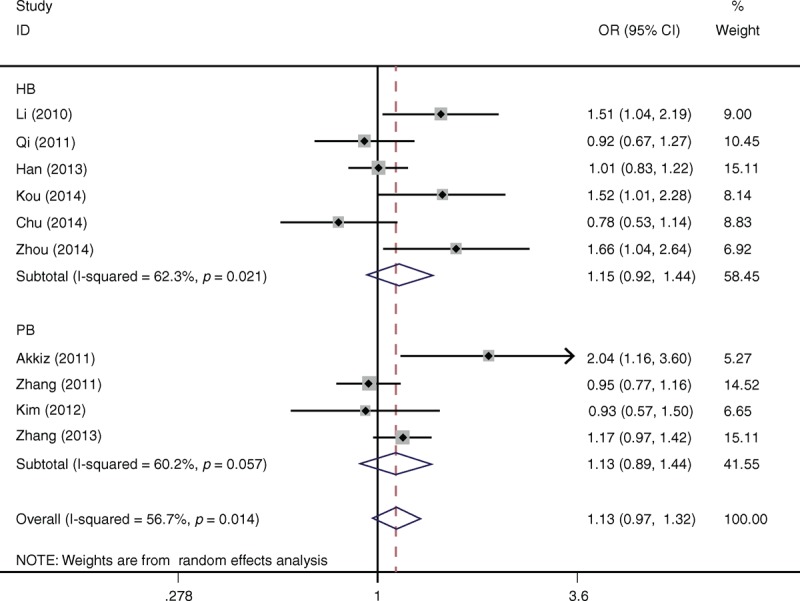
Forest plots of miR-196a2 rs11614913 polymorphism and HCC risk in subgroup analysis by source of controls using a random-effect model (dominant model CC+TC vs TT).

**FIGURE 3:**
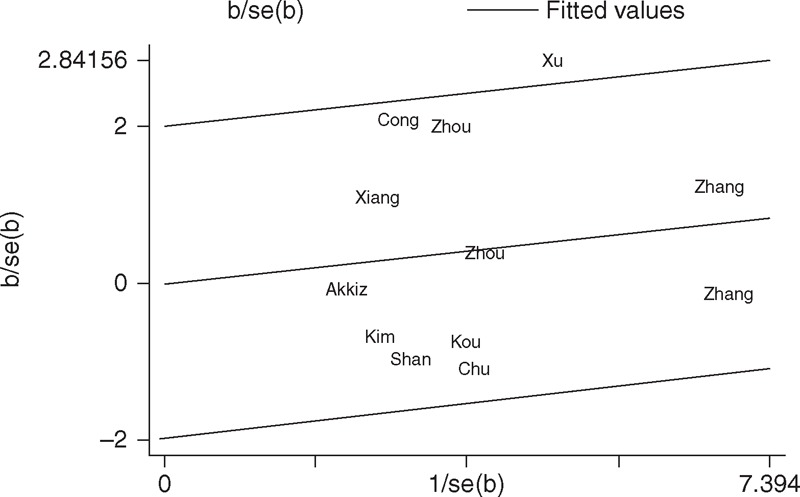
Galbraith plots of miR-146a rs2910164 polymorphism and HCC risk (homozygote GG vs CC). The study of Xu et al was spotted as the outlier.

Table 3 lists the main results of meta-analysis of miR-196a2 rs11614913 polymorphism and HCC risk. There was no evidence of significant association between miR-196a2 rs11614913 polymorphism and HCC risk when all eligible studies were pooled into the meta-analysis (CC vs TT: OR = 1.287, 95% CI: 0.931–1.607, *P* = 0.226; TC vs TT: OR = 1.055, 95% CI: 0.958–1.161, *P* = 0.278; CC+TC vs TT: OR = 1.134, 95% CI: 0.974–1.320, *P* = 0.105, Figure 3; CC vs TC+TT: OR = 1.203, 95% CI: 0.936–1.398, *P* = 0.136). In subgroup analyses by ethnicity, source of controls, and HWE status, statistical significant association was also not detected in all subgroups (Figure 2).

**Table 3:**
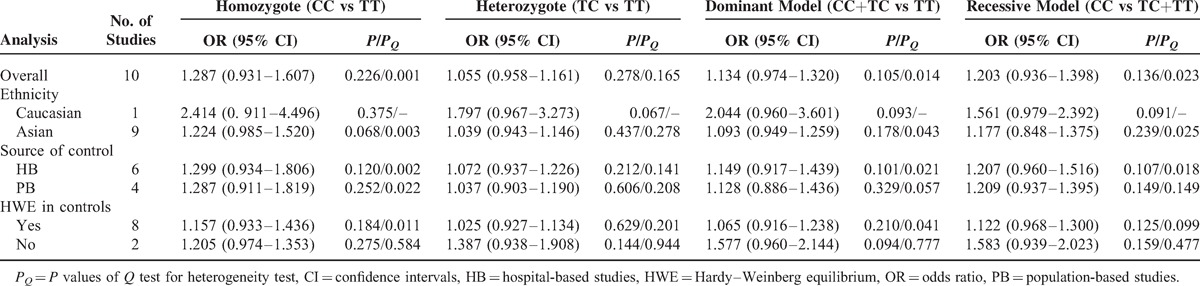
Meta-Analysis of miR-196a2 rs11614913 Polymorphism and HCC Risk

### Heterogeneity Analysis

With respect to miR-146a rs2910164 polymorphism, the *P*_*Q*_ value of the *Q* test was <0.10 in codominant model in the overall populations (GG vs CC: *P*_*Q*_ = 0.081), indicating statistical significant heterogeneity among studies. To investigate the sources of heterogeneity, we performed metaregression and subgroup analyses. Metaregression analysis revealed that the ethnicity, genotyping methods, source of controls, and HWE in controls were not effect modifiers. Subsequently, we performed subgroup analyses by ethnicity, source of control, and HWE in controls. However, heterogeneity still existed in codominant model (GG vs CC) in Asians and hospital-based studies (Table 2). To further explore the source of heterogeneity, we performed Galbraith plots analysis to identify the outliers which might contribute to the heterogeneity. Our results revealed that the study Xu et al^15^ was the outlier in the overall populations (Figure 3). All *P*_*Q*_ values were >0.10 after excluding the study Xue et al^15^ in the overall populations, Asians and hospital-based studies. Interestingly, the significance of the pooled ORs for the miR-146a rs2910164 polymorphism and HCC risk in the overall populations, Asians and hospital-based studies were not influenced by excluding this study.

**FIGURE 4:**
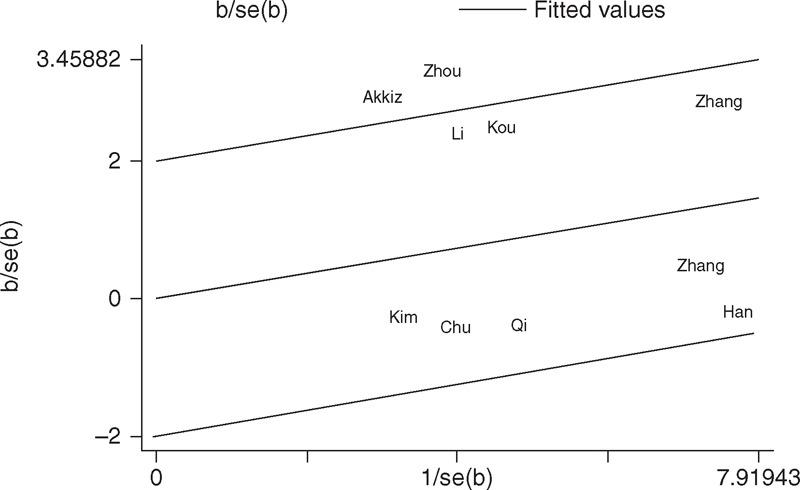
Galbraith plots of miR-196a2 rs11614913 polymorphism and HCC risk (homozygote CC vs TT). The studies of Akkiz et al and Zhou et al were spotted as the outliers.

With respect to miR-196a2 rs11614913 polymorphism, statistical significant between-study heterogeneity was also found in the pooling analyses of total eligible studies (CC vs TT: *P*_*Q*_ = 0.081; CC+TC vs TT: *P*_*Q*_ = 0.014; CC vs TC+TT: *P*_*Q*_ = 0.016; Table 3). Metaregression analysis indicated that the source of control was the major source heterogeneity. The ethnicity, genotyping methods, and HWE in controls were not effect modifiers. Galbraith plots analysis suggested that the studies by Zhou et al^27^ and Akkiz et al^34^ were the outliers and the major source of the heterogeneity (Figure 4). The *P*_*Q*_ values were greater than 0.10 after excluding the two studies Zhou et al^27^ and Akkiz et al^34^ in the overall populations, Asians, hospital-based studies, and studies consistent with HWE. However, the significance of the pooled ORs for the miR-196a2 rs11614913 polymorphism and HCC risk in the overall populations, Asians, hospital-based studies, and studies consistent with HWE were also not changed by excluding the two studies.

### Sensitivity Analysis

Sensitivity analysis was carried out by sequential omission of individual studies for both the miR-146a rs2910164 and miR-196a2 rs11614913 polymorphisms. For analyses of pooling more than three studies, the significance of ORs was not influenced materially by omitting any single study (data not shown). For the miR-146a rs2910164 polymorphism, sensitivity analysis was further performed by omitting the studies by Shan et al, Kou et al, and Zhou et al^23,24,27^ in which the control populations were not in accordance with HWE. The significance of all ORs was not altered after excluding these 3 studies. For the miR-196a2 rs11614913 polymorphism, sensitivity analysis was also performed by excluding those 2 studies by Kou et al and Zhou et al^24,27^ in which the control populations were deviated from HWE, and the significance of all ORs was also not altered.

### Publication Bias

**FIGURE 5:**
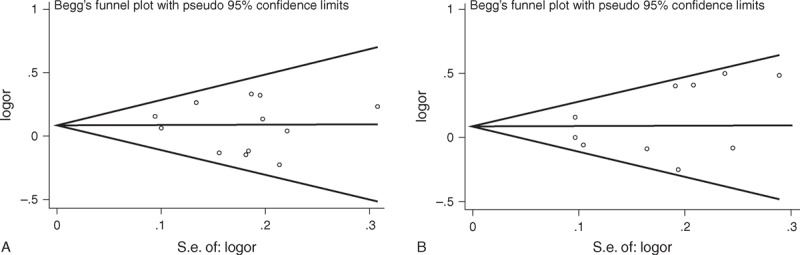
Funnel plot analysis to detect publication bias. Each point represents a single study for the indicated association. (A) Funnel plot for miR-146a rs2910164 polymorphism in overall analysis (dominant model GG+CG vs CC: P = 0.453). (B) Funnel plot for miR-196a2 rs11614913 polymorphism in overall analysis (dominant model CC+TC vs TT: P = 0.621).

Begg's funnel plot and Egger's test were used to access the publication bias in this study. Funnel plots shapes did not show obvious evidence of asymmetry (Figure 5), and all the *P* values of Egger's tests were greater than 0.05 for both the miR-146a rs2910164 and miR-196a2 rs11614913 polymorphisms, providing statistical evidence of the funnel plots’ symmetry. The results suggested that publication bias did not exist in this study.

## DISCUSSION

MicroRNAs, as a family of endogenous small single-stranded non-coding RNAs, participate in the regulation of diverse biological functions, such as cell proliferation, differentiation, apoptosis, genome rearrangements, and transcriptional regulations.^37,38^ Mounting evidence has demonstrated that miRNAs are essential for normal development and cellular homeostasis and thereby, dys-functions in these molecules have been associated with human cancers including HCC.^39^ SNPs or mutations occurring in the miRNA gene region may affect the property of miRNAs through altering miRNA expression and/or maturation. MiR-146a rs2910164 and miR-196a2 rs11614913 were 2 most common studied SNPs in miRNA. To date, many epidemiological studies have been conducted to investigate the association of miR-146a rs2910164 and miR-196a2 rs11614913 polymorphisms with HCC risk, but the results remain controversial and underpowered. Therefore, we performed this meta-analysis including all available studies to provide the most comprehensive assessment of the associations. Our results based on 22 studies suggested that the miR-146a rs2910164 polymorphism was significantly associated with increased HCC risk. However, our data did not support a genetic association of the miR-196a2 rs11614913 polymorphism with HCC susceptibility.

MiR-146a rs2910164 polymorphism is located in the 3p strand of miR-146a with a C to G change in the passenger strand and involves a mispairing in the hairpin of the precursor, which leads to altered processing and lower expression of the mature sequence.^40^ It was reported that the G allele of the rs2910164C>G polymorphism within the miR-146a sequence reduced the production of mature miR-146a compared with the G allele.^41^ Moreover, The G allele also interfered with the binding capacity of a nuclear factor to pre-miR-146a. The reduction in miR-146a led to less-efficient inhibition of the target genes involved in the Toll-like receptor and cytokine signaling pathway (TRAF6, IRAK1), deregulation of which is possibly involved in the carcinogenesis including HCC. Therefore, it is biologically plausible that the miR-146a rs2910164 polymorphism plays a critical role in hepatocarcinogenesis. This hypothesis was confirmed by our meta-analysis.

In the present study, we observed that the miR-146a rs2910164 polymorphism presented a risk factor for HCC in Asians, but not in Caucasians. The inconsistent results among diverse ethnicities demonstrated different effects of the miR-146a rs2910164 polymorphism on HCC risk in different ethnic genetic backgrounds. Nevertheless, because of the small number of eligible studies among Caucasians included in this study, the detected association between the miR-146a rs2910164polymorphism and HCC risk in Caucasians is likely to be caused by chance, because study with small sample sizes may have insufficient statistical power to determine a slight effect or may have generated a fluctuated estimation. In the present study, there was only one study for Caucasians concerning the miR-146a rs2910164 polymorphism on HCC risk.^11^ Therefore, the results from the Caucasian population should be interpreted with caution. Additional studies are warranted to further validate the ethnic differences in the effect of this functional polymorphism on HCC risk, especially in Caucasians.

Previous studies demonstrated that the hospital-based studies have inherent selection bias because of the fact that the controls may just represent a sample of ill-defined reference population, and may not be representative of the study population or the general population, particularly when the genotypes under investigation were associated with the disease-related conditions that the hospital-based controls may have.^42^ Selecting of proper and representative population-based controls is of great value in eliminating biases in such genotype association studies. Therefore, we performed subgroup analysis stratified by source of control. The results revealed that the miR-146a rs2910164 polymorphism was associated with an increased HCC risk in population-based studies, but not in hospital-based studies. Therefore, a methodologically preferable design, such as using a proper and representative population-based high quality study, is of great importance in case–control studies.

It has been widely accepted that the studies disobeyed the law of HWE may be as a result of genetic reasons including non-random mating, or the alleles reflect recent mutations that have not reached equilibrium, as well as methodological reasons including biased selection of subjects from the population or genotyping errors.^43^ Because of the reasons of disequilibrium, the results of genetic association studies might be spurious if the distribution of genotypes in the control groups were inconsistent with HWE. In the present study, the genotype distributions of the controls in 3 studies were inconsistent with HWE for miR-146a rs2910164 polymorphism.^23,24,27^ Hence, we carried out subgroup analysis by HWE in controls. When excluding the studies that were inconsistent with HWE, the results were persistent and robust, suggesting that this factor probably had little effect on the overall estimates.

One of the major concerns in a sound meta-analysis is the degree of heterogeneity that exists between the studies because heterogeneous data are liable to result in misleading results, and finding the sources of heterogeneity is one of the most important goals in a meta-analysis.^44^ In the present study, significant between-study heterogeneity in the pooled analyses of total eligible studies was observed. To find the sources of heterogeneity, we performed subgroup analyses and metaregression. Subgroup analyses revealed that the heterogeneity was still significant in most of the subgroups for both the miR-146a rs2910164 and miR-196a2 rs11614913 polymorphisms. Metaregression analysis showed that none of the investigated covariates was the source of heterogeneity for miR-146a rs2910164 polymorphism, while the source of control was the major source heterogeneity for miR-196a2 rs11614913 polymorphism. Subsequently, we performed Galbraith plots to further investigate the heterogeneity. For the miR-146a rs2910164 polymorphism, Galbraith plots spotted 1 study^15^ as the outlier and the possible source of heterogeneity. For the miR-196a2 rs11614913 polymorphism, Galbraith plots spotted 2 studies^27,34^ as the outliers and the possible source of heterogeneity. When excluding the study by Xu et al^15^ for miR-146a rs2910164 polymorphism and the studies by Zhou et al^27^ and Akkiz et al^34^ for miR-196a2 rs11614913 polymorphism, all *P*_*Q*_ values were greater than 0.10 in all genetic comparison models in the overall populations and all subgroups. Interestingly, the summary ORs for the miR-146a rs2910164 and miR-196a2 rs11614913 polymorphisms in different comparison models in the overall population and subgroup analyses were not material changed by excluding the outlier studies, suggesting that our results were robust and reliable.

Some limitations of this study should be addressed. First, the controls in the eligible studies were not uniformly defined. Although the controls were selected mainly from healthy subjects, some had benign disease such as liver cirrhosis, HBsAg positive and so on. Therefore, non-differential misclassification bias was possible because these studies may have included the control populations who have different risks of developing HCC. Second, in subgroup analysis by ethnicity, the included studies regarded only Asians and Caucasians. Data concerning other ethnicities were not observed. Therefore, additional studies are needed to evaluate the effect of the two SNPs on HCC risk in different ethnicities. Third, the results were based on unadjusted estimates. We did not perform analysis adjusted for other covariates such as sex, age, drinking and smoking status, HBV and HCV carrier status, environment factors, and so on, because of the absence of original data in the eligible studies.

Despite the limitations, the results suggest that the miR-146a rs2910164 polymorphism contributes to increased HCC susceptibility, especially in Asian populations. Further large and well-designed studies are required to validate this association.
